# Free radical scavenging, *α-*glucosidase inhibitory and lipase inhibitory activities of eighteen Sudanese medicinal plants

**DOI:** 10.1186/s12906-018-2346-y

**Published:** 2018-10-19

**Authors:** Sara Mustafa Idris Elbashir, Hari Prasad Devkota, Mikiyo Wada, Naoki Kishimoto, Masataka Moriuchi, Tsuyoshi Shuto, Shogo Misumi, Hirofumi Kai, Takashi Watanabe

**Affiliations:** 1 Department of Medicinal Botany, Graduate School of Pharmaceutical Sciences, Kumamoto University, 5-1 Oe-honmachi, Chuo-ku, Kumamoto, Kumamoto 862-0973 Japan; 2 Program for Leading Graduate Schools, Health Life Science: Interdisciplinary and Glocal Oriented (HIGO) Program, Kumamoto University, Kumamoto, Japan; 3 School of Pharmacy, Kumamoto University, 5-1 Oe-honmachi, Chuo-ku, Kumamoto, Kumamoto 862-0973 Japan; 4 Department of Environmental and Molecular Health Sciences, Faculty of Medical and Pharmaceutical Sciences, Kumamoto University, Kumamoto, 862-0973 Japan; 5 Department of Molecular Medicine, Faculty of Medical and Pharmaceutical Sciences, Kumamoto University, Kumamoto, 862-0973 Japan

**Keywords:** Diabetes, Sudan, Medicinal plants, Antioxidant, *α*-Glucosidase, Pancreatic lipase

## Abstract

**Background:**

Lifestyle-related diseases such as diabetes are steadily increasing worldwide. In Sudan, there are a variety of plant species used traditionally for the treatment of diabetes, obesity and other symptoms which need to be validated through scientific studies for their claimed traditional uses. Therefore, in the current study, the free radical scavenging activity, *α*-glucosidase inhibitory and pancreatic lipase inhibitory activities of 70% ethanol and water extracts of eighteen Sudanese medicinal plants were investigated using various in vitro assays. Moreover, the cytotoxicity and genotoxicity were assessed for the bioactive plant extracts.

**Methods:**

Eighteen plants were selected on the basis of their traditional uses and extracted with 70% ethanol and water to obtain thirty-six extracts. The obtained extracts were screened using different in vitro bioassays namely, 1,1-diphenyl-2-picrylhydrazyl (DPPH) radical scavenging, *α*-glucosidase inhibitory and pancreatic lipase inhibitory assays. Furthermore, the active plant extracts were investigated for their cytotoxicity and genotoxicity on HeLa cell line using HCS DNA Damage Assay.

**Results:**

Both 70% ethanol and water extracts of *Acacia nilotica, Ziziphus spina-christi, Abrus precatorius,* and *Geigeria alata* along with the 70% ethanol extract of *Martynia annua* showed potent free radical scavenging activity. Regarding the *α*-glucosidase inhibition assay, both extracts of *Acacia nilotica*, *Ziziphus spina-christi*, *Geigeria alata,* and *Cyperus rotundus* showed potent activity. In general, 70% ethanol extracts were more potent compared to water extracts with exception of *Cordia sinensis* and *Cymbopogon proximus,* for which water extracts also showed potent enzyme inhibitory activity. Similarly, water extracts of *Acacia nilotica* and *Ziziphus spina-christi* showed potent inhibitory activity against pancreatic lipase enzyme. Some of the extracts also showed significant genotoxicity and cytotoxicity at the concentration range used for bioactivities.

**Conclusion:**

The extracts of *Acacia nilotica, Ziziphus spina-christi, Geigeria alata, Martynia annua* and *Abrus precatorius* exhibited an appreciable range of activity on antioxidant and enzyme inhibitory assays.

## Background

Diabetes mellitus (DM) is a serious chronic metabolic disorder, with life-threatening complications. The condition is defined by a state of chronically elevated blood glucose levels (hyperglycemia) with disturbances of carbohydrate, fat and protein metabolism, and mainly caused by deficiency or diminished effectiveness of endogenous insulin [1]. DM is an important cause of mortality and morbidity globally and has considerable health-system costs consequences. According to the World Health Organization (WHO), the total number of people with DM worldwide has substantially increased between 1980 and 2014, rising from 108 million to current numbers of 422 million, which are around four times higher [2]. This rise in the burden of the disease is greatly observed in the middle- and low-income countries compared to high-income countries [3]. In 2017, the International Diabetes Federation (IDF) estimated that 9.6% adults aged 20–79 years are living with DM in the Middle East and Northern Africa region, about 49.1% of them are still undiagnosed and 83.8% are living in low or middle-income countries, such as Sudan [4].

In Sudan, non-communicable diseases (NCD), including diabetes mellitus have become the principal cause of morbidity and mortality, with an overall prevalence of 10.9% among adults, according to the IDF [4, 5] . Furthermore, DM accounts for about 6% of total deaths from non-communicable diseases in Sudan [6]. In spite of that, diabetes care in Sudan is still very poor to achieve proper glycemic goals and a good quality of life for diabetic patients [7]. According to a recent study conducted in Khartoum state, Sudan, the main factors of the insufficient diabetes services at primary health care centers are the lack of the follow up system for diabetic patients, the insufficient investigations, the lack of diet therapy guidelines and poor patient knowledge and behavior towards the disease management [8]. Moreover, treatment with the current available antidiabetic drugs is still not satisfactory to rectify a normal pattern of glycemic control, and unable to prevent the long-term diabetic complications as well [9]. Therefore, the need for effective, safe and affordable alternative treatment options has become crucial. As an alternative source of medicine, medicinal plants and herbal remedies have always gained a growing public interest across the globe. In Sudan, the use of medicinal plants was always inherent in Sudanese traditional medicine. People in many areas of the country, especially in rural areas, depend mainly on herbal medicine to cure their health problems as they believe in their safety, efficacy, besides to their availability and affordability for a huge sector of the population [10]. One important property of medicinal plants associated with DM treatment is antioxidant activity. There are several scientific reports and clinical evidence that the oxidative stress condition associated with the generation of reactive oxygen species (ROS) increases in both type 1 and type 2 diabetes [11]. It is believed to play an important role in the development of DM vascular complications through endothelial dysfunction and inflammation [12]. In addition, it has been suggested that the cause of the vascular complications in diabetic patients can be due to the insufficient response of the cellular antioxidants against the oxidative stress induced by hyperglycemia [13]. In this context, medicinal plants with potential antioxidant activity may be of great benefit to diminish oxidative stress in diabetic patients and profoundly contribute to reducing the severity and complications of the disease. On the other hand, obesity is a worldwide health problem, which is strongly associated with a number of chronic diseases such as DM [14]. Inhibition of nutrients digestion and absorption is an important target for the treatment of obesity and related diseases like DM. Pancreatic lipase and *α*-glucosidase enzymes inhibitors can lower blood glucose levels by reducing the intestinal absorption of fats and carbohydrates, respectively [15, 16].

Fortunately, the flora of Sudan has an immense diversity of indigenous plant species, owing to the fact that they extend over six agro-ecological zones, including desert and semi-desert in the northern part, high and low rainfall savannah to the south, mountain region and flood zone. Several floristic studies revealed that a total of 3137 species of flowering plants representing 170 families exist in Sudan [17]. More than 136 species of them are used commonly as medicinal plants in Khartoum State, from which, 90 plant species indigenous to Sudan [18]. Previous studies have reported a considerable range of biological activities of Sudanese medicinal plants, such as antibacterial [19, 20], anthelmintic [21], antiparasitic, antiplasmodial [22], anticancer [23], antidiabetic [24], and many other activities. However, many other Sudanese medicinal plants did not receive any test for their efficacy to validate their claimed traditional uses. In a different study, we screened six medicinal plants from Sudan for their antioxidant and enzyme inhibitory activities using different extraction solvents, and found that the water and aqueous ethanol extracts were suitable solvents for extraction of phenolic compounds [25]. In this context, in the present study, thirty-six extracts derived from 18 plant species belonging to 14 families collected from North Kurdufan and Khartoum states, Sudan (Table 1) were investigated for their potential antioxidant, *α*-glucosidase inhibitory and pancreatic lipase inhibitory activities. The adverse effects and toxicity due to the presence of genotoxic and cytotoxic agents in medicinal plants is important from the point of view of their therapeutic application [26]. Therefore, ten  active extracts in antioxidant and enzyme inhibitory assays were further investigated for their potential genotoxicity and cytotoxicity on HeLa cells using HCS DNA Damage assay.

**Table 1:** Scientific name, family, parts used, local name, traditional use and voucher specimen number of species under study

No.	Scientific name	Family	Parts used	Local name (Sudanese accent)	Traditional use	Voucher specimen no.
1	*Abrus precatorius* L.	Fabaceae	Seeds	Habbat Al-Arus	Used in the treatment of diabetes mellitus, sterility, inflammation of the eye, headache, and as a laxative, purgative, anti-cough, emetic, demulcent, and as an agent in water purification [10].	MAPRI-H.G.117/83
2	*Acacia nilotica* L.	Fabaceae	Pods	Garad, Sunt	Used in the treatment of hypertension, diabetes mellitus, cough and as a gargle for tonsillitis [10, 48].	MAPRI-H.G.112/83
3	*Blepharis linariifolia* Pers*.*	Acanthaceae	Whole plant	Al-Bighail; Shoak Al-Dhab, and Siha	Used in the treatment of urinary disorders (Kidney stones), as a general tonic, and for stomach pain [49].	MAPRI-H.M.138/77
4	*Boswellia papyrifera* (Del.) Hochst.	Burseraceae	Gum	Tarag tarag, Shagar El-luban	Used in the treatment of dysentery, upper respiratory tract infections, and as an ingredient of a special Sudanese incense [17, 50].	MAPRI-H.K.12/96
5	*Cephaelis ipecacuanha* (Brot.) A.Rich	Rubiaceae	Roots	Irg Al-dahab	Used in the treatment of dysentery and as an emetic [51].	MAPRI-H.K.19/53
6	*Citrullus colocynthis* L.	Cucurbitaceae	Seeds	Handal	Used in the treatment of diabetes mellitus, gonorrhea, anti-rheumatic, and against scabies. It is also used for making tar (qutran), against moth, lice, and as anti-scorpion stings and anti-snake bite [10, 17].	MAPRI-H.O.5/78
7	*Cordia sinensis Lam*.	Boraginaceae	Leaves	Andrab	Used in the treatment of diabetes, wound, high fever and as antitumor [52, 53].	MAPRI-H.O.22/78
8	*Cymbopogon proximus* (Hochst. ex A. Rich) Stapf.	Poaceae	Whole plant	Mahareb	Used in the treatment of kidney infections, diabetes, gout, renal colic, prostatic enlargement. It also used as carminative, antispasmodic, and diuretic [17, 54].	MAPRI-H.12.78
9	*Cyperus rotundus* L*.*	Cyperaceae	Roots	Si’da	Used in the treatment of diabetes, abdominal pain, diarrhea, as anthelmintic, astringent, and aromatic [55].	MAPRI-H.ABU.H.16/94
10	*Geigeria alata* DC.	Asteraceae	Aerial parts	El Gadgad	Used in the treatment of diabetes mellitus, antihypertensive, and intestinal complaints, and cough. It is also used as antispasmodic [42, 56].	MAPRI-H.G.61/93
11	*Martynia annua* L.	Martyniaceae	Mature Fruit	Gara Gebei	Used as anthelmintic, antibacterial, analgesic, anti-venom, antipyretic, anti-convulsion, antidiabetic and for wound healing [41].	MAPRI-H.Y.22/14
12	*Pennisetum glaucum* (L.) R.Br.	Poaceae	Grains	Dukhun	Used in the treatment of rheumatism and as a food additive [10, 57].	MAPRI-H.Y.53/010
13	*Ruta graveolens* L.	Rutaceae	Fruit	Sathab	Used in the treatment of rheumatic pains [10].	MAPRI-H.Y.7/012
14	*Solenostemma argel* Del.	Apocynaceae	Leaves	Harjal	Used in the treatment of diabetes mellitus, epigastric pain, joints affections, fever, common cold, headache, loin pain, puerperal fever, nausea, indigestion. Its beverage is used as laxative, purgative, carminative, and anti-spasmodic [10, 17, 58].	MAPRI-H.O.12/78
15	*Tinospora bakis* Miers	Menispermaceae	Roots	Erg El-Haggar	Used in the treatment of diabetes mellitus, headache, and abdominal pain. [48, 59].	MAPRI-H.M.30/76
16	*Trigonella foenum-graceum* L.	Fabaceae	Seeds	Hilba	Used in the treatment of diabetes mellitus, abdominal disorders, dysentery, and, as anti-spasmodic, antidiarrheal, anti-amoeba. It is also used as a food additive and to increase secretion of milk for lactating mothers [17, 60].	MAPRI-H.Y.6/005
17	*Vangueria madagascariensis* J.F.Gmel.	Rubiaceae	Fruit	El-Kirkir	Used in the treatment of diabetes mellitus and bacterial infections [61].	MAPRI-H.W.45/95
18	*Ziziphus spina-christi* L.	Rhamnaceae	Roots	Sidir	Used in the treatment of gonorrhea, fever, as anti-spasmodic and anti-purgative [17, 62].	MAPRI-H.Y.4/010

## Methods

### Plant materials

Eighteen plant species (Table 1) were collected from North Kurdufan and Khartoum states, Sudan, on March 2016. The target plant parts were separated, identified morphologically, compared with identified herbarium specimens and dried at 60 °C using an electric oven. All plant samples were authenticated by Mr. Yahya Sulieman Mohamed, Taxonomist, Herbarium of Medicinal, Aromatic Plant and Traditional Medicine Research Institute (MAPTRI), Khartoum, Sudan, and deposited in the herbarium of the Institute. Voucher specimen numbers for these plant species are given in Table 1.

### Chemicals and equipment

6-Hydroxy-2,5,7,8-tetramethylchroman-2-carboxylic acid (Trolox), 1,1-diphenyl-2-picrylhydrazyl (DPPH), dimethylsulfoxide (DMSO), disodium hydrogenphosphate 12-water, sodium dihydrogenphosphate dehydrate, Triton® X-100, and Valinomycin were purchased from Wako Pure Chemical Industries, Ltd., Osaka, Japan. 4-Methylumbelliferyl oleate (4-MUFO) was purchased from Toronto Research Chemicals, Toronto, Ontario, Canada. Acarbose, *p*-nitrophenyl glucopyranoside (PNGP) and di-potassium hydrogenphosphate were purchased from Nacalai Tesque, Inc., Kyoto, Japan. *α*-Glucosidase (from *Saccharomyces cerevisiae*)*,* porcine pancreatic lipase (PPL, type II), and bovine serum albumin (BSA) were purchased from Sigma-Aldrich Co. (St. Louis, MO, USA). Cetilistat was purchased from Combi-Blocks, Inc., Japan. Potassium dihydrogen phosphate was purchased from Kanto Chemical Co., Inc. Tokyo, Japan, and 2-morpholinoethanesulfonic acid, monohydrate (MES) was purchased from Dojindo Chemical Research, Kumamoto, Japan. Paraformaldehyde was purchased from Chiyoda Inc. and HCS DNA Damage kit was purchased from Thermo Fisher Scientific Inc.

Absorbance was recorded on the Infinite 200 PRO® (Tecan Austria GmBH, Grodig, Austria) spectrophotometer (for DPPH and *α*-Glucosidase inhibitory assays) and Wallac ARVO™ MX 1420 multilabel counter spectrophotometer (Perkin Elmer Japan, Kanagawa, Japan) (for lipase inhibitory assay).

### Preparation of plant extract

About 20 g of each plant sample was ground and extracted with two different solvents (70% aqueous ethanol and water) by the use of ultra-sonication assisted extraction at 50 °C for two hours. Both extracts were then filtered. 70% Ethanolic extracts were concentrated and evaporated under reduced pressure using rotatory evaporator. Whereas, water extracts were freeze-dried.

### DPPH free radical scavenging activity

The free radical scavenging activity was measured according to the modified method of Shimamura et al. [27], One hundred μL of sample extract (in 50% (*v*/v) ethanol) with varying concentrations were mixed with 50 μL of 200 mM MES buffer (pH 6.0) and 50 μL of 800 μM DPPH in 99.5% ethanol solution. After that the assay plate was kept in dark at room temperature for 20 min, then the absorbance was measured at 520 nm. Trolox was used as a positive control and the percentage radical scavenging capacity (SC%) of samples was obtained from the following equation:

SC% = [(AC-A0)-(AS-A0)/ (AC-A0)] × 100, where AC is the absorbance of the control, which contained all reagents except test sample, A0 is absorbance of the blank which contained the sample and all reagents except DPPH and AS is the absorbance of the test samples. From these data, the SC_50_ value was calculated, which is defined as the concentration of the extract required for 50% reduction of the DPPH radical absorbance. All samples were assayed in triplicate.

### *α*-Glucosidase inhibitory activity

*α*-Glucosidase inhibitory activity was measured according to the method by Yang et al. [28] with slight modifications. Briefly, 10 μL of the sample solution with varying concentrations was mixed with 60 μL of phosphate buffer (200 mM pH 6.8) and 10 μL of *α*-glucosidase (1 U/ml) in phosphate buffer. After incubation at 37 °C for 5 min, 20 μL of PNGP (4 mM) was added to start enzyme reaction. The absorbance of the reaction solutions was measured right after the addition of substrate for 12 min at 405 nm. Acarbose was used as the positive control. The percentage inhibition of *α*-glucosidase activity was calculated via the following equation:

% inhibition = [1-(AS at 12 min − AS at 0 min)/ (AC at 12 min − AC at 0 min)] × 100, where AS is the absorbance readings of samples, AC is the absorbance readings of the control which contain all solvents except the sample. The concentration of the tested samples giving 50% inhibition of the enzyme activity (IC_50_) was estimated from the plots of the concentration vs. the inhibitory activity, all samples were assayed in triplicate.

### Lipase inhibitory activity

Porcine pancreatic lipase inhibitory activity was measured according to the modified method of Bitou et al. [29], the assay was performed using 96-well microplate. Briefly, the reaction mixture was prepared with 50 μL of porcine pancreatic lipase (50 U/ml) dissolved in phosphate buffer (200 mM pH 7.4), and 50 μL sample solution with varying concentrations and left at room temperature for 10 min. The reaction was started by adding 100 μL of 0.5 mM 4-MUFO diluted in phosphate buffer (0.2 M pH 7.4) from 0.1 M stock solution in DMSO. The amount of 4-methylumbelliferone released by enzyme reaction was measured fluorometrically at an excitation wavelength of 355 nm and an emission wavelength of 460 nm for 8 min. Cetilistat was used as the positive control. The relative lipase inhibition activity was calculated using the following formula:

% Inhibition = [1-(FS at 8 min − FS at 0 min)/ (FC at 8 min − FC at 0 min)] × 100, where FS is the fluorescence readings of samples which contain all solvents and sample and FC is the control which contains all solvent but the sample. The concentration of the tested samples giving 50% inhibition of the enzyme activity (IC_50_) was estimated from the plots of the concentration vs. the inhibitory activity, all samples were assayed in triplicate.

### Cell culture

The HeLa cell line was cultured in Dulbecco’s Modified Eagle’s medium (DMEM) supplemented with 10% fetal calf serum (FCS). The cells were maintained at 37 °C in a CO_2_ incubator with 90% humidity and 5% CO_2_. Cells were subcultured regularly using trypsin/EDTA.

### Cell genotoxicity/cytotoxicity assay using the HCS DNA damage kit

HeLa cells were seeded at a density of 7500 cells/100 μL /well on 96-well plate. After 24 h incubation, 100 μL of the plant extracts with different concentrations was added to cells along with the control drug (Valinomycin) and incubated for 24 h. Treated cells were then stained by adding 50 μL of Image-iT® Dead Green™ viability stain and incubated for 30 min. Then the medium was removed and cells were fixed, permeabilized and blocked by incubation with 4% paraformaldehyde, Triton® X-100 in PBS and bovine serum albumin (BSA) in PBS solutions, respectively. Cells were then incubated with 50 μL of pH2AX mouse monoclonal primary antibody solution for 1 h at room temperature. After washing in PBS solution three times, Cells were further incubated with 50 μL of Alexa Fluor® 555 goat anti-mouse IgG (H + L)-conjugated secondary antibody and Hoechst 33342 nuclear counterstain solution for 1 h in dark. Then washed with PBS solution three times and 100 μL of PBS was added. Stained cells were observed using Keyence BZ-X700 (Keyence, Japan).

### Statistical analysis

For quantitative analysis, the result represents the mean ± SD (*n* = 3) and the data were analyzed using the Scheffe’s test by a Statview software as indicated in each figure legend. The level of significance was set at *p* < 0.05.

## Results

### Extraction yield

The extraction yield (%) of the 18 Sudanese medicinal plants using 70% ethanol and water as solvents is shown in Table 2.

**Table 2:** Percentage extraction yield of the plants included in the study

Scientific name	Extraction yield (%)	
	70% Ethanol extract	Water extract
*A. precatorius*	9.6	11.8
*A. nilotica*	27.9	29.2
*B. linariifolia*	7.6	8.0
*B. papyrifera*	34.3	33.2
*C. ipecacuanha*	10.6	11.4
*C. colocynthis*	4.1	9.8
*C. sinensis*	11.4	12.2
*C. proximus*	5.8	5.0
*C. rotundus.*	5.5	2.2
*G. alata*	10.1	11.6
*M. annua*	2.4	2.4
*P. glaucum*	5.1	5.4
*R. graveolens*	24.3	21.6
*S. argel*	20.2	15.0
*T. bakis*	9.4	8.9
*T. foenum-graceum*	13.7	11.8
*V. madagascariensis*	28.5	35.8
*Z. spina-christi*	19.9	16.6

% extraction yield is expressed as *w*/w g of dried extract

### Free radical scavenging activity

In the present study, DPPH radical scavenging assay was used to evaluate the in vitro antioxidant activity for all plant extracts. Results are presented as SC_50_, the lower SC_50_ values indicate the stronger antioxidant activity (Table 3). Trolox, a water-soluble derivative of vitamin E was used as positive control, with an SC_50_ value of 11.35 ± 0.05 μg/ml. These results showed that both extracts of *A. nilotica* exhibited the highest antioxidant activity among extracts. Furthermore, 70% ethanol and water extracts of *A. nilotica* were more active than the positive control Trolox with SC_50_ values of 4.06 ± 0.09 and 7.51 ± 0.19 μg/ml, respectively. Approximately, similar scavenging activity as the positive control was shown by the ethanolic extracts of *Z. spina-christi* and *A. precatorius,* with SC_50_ values of 10.75 ± 0.08 and 13.66 ± 0.12 μg/ml, respectively. For the ethanolic extracts of *M. annua* and *G. alata*, the scavenging ability was moderate compared to the positive control, with SC_50_ values of 31.65 ± 0.69 and 73.68 ± 3.11 μg/ml, respectively. On the other hand, the free radical scavenging activity of the water extracts of *A. precatorius*, *Z. spina-christi* and *G. alata*, was less potent with SC_50_ values of 20.93 ± 3.54, 36.01 ± 3.60 and 137.66 ± 1.31 μg/ml, respectively.

**Table 3:** DPPH radical scavenging activity of extracts

Scientific name	SC_50_ (μg/ml)^a^ value for DPPH radical scavenging activity	
	70% Ethanol extract	Water extract
*A. precatorius*	13.66 ± 0.12	20.93 ± 3.54
*A. nilotica*	4.06 ± 0.09	7.51 ± 0.19
*B. linariifolia*	105.05 ± 2.59	336.73 ± 4.49
*B. papyrifera*	120.53 ± 3.09	301.53 ± 0.48
*C. ipecacuanha*	251.28 ± 4.57	359.18 ± 2.89
*C. colocynthis*	507.67 ± 8.69	698.20 ± 9.68
*C. sinensis*	316.24 ± 8.83	301.5 ± 10.91
*C. proximus*	201.88 ± 0.03	333.99 ± 3.54
*C. rotundus.*	142.23 ± 5.88	270.64 ± 9.62
*G. alata*	73.68 ± 3.11	137.66 ± 1.31
*M. annua*	31.65 ± 0.69	281.94 ± 2.65
*P. glaucum*	127.84 ± 5.14	257.19 ± 2.08
*R. graveolens*	187.24 ± 1.50	362.04 ± 0.93
*S. argel*	334.35 ± 6.01	411.42 ± 7.03
*T. bakis*	278.04 ± 3.92	438.37 ± 2.99
*T. foenum-graceum*	285.58 ± 2.22	455.84 ± 4.69
*V. madagascariensis*	463.87 ± 5.21	552.27 ± 11.86
*Z. spina-christi*	10.75 ± 0.08	36.01 ± 3.60
Trolox^b^	11.35 ± 0.05	

^a^Each value represents the mean ± SD (*n* = 3)

^b^Trolox was used as a positive control

### *α*-Glucosidase inhibitory activity

In this study, we aimed to explore *α*-glucosidase inhibitors of plant origin. Thus, we investigated the inhibitory effect of thirty-six ethanolic and water extracts on *α-*glucosidase enzyme obtained from *Saccharomyces cerevisiae*. Table 4 shows the results of the in vitro assay of *α*-glucosidase inhibitory activity for all extracts. Acarbose was used as a positive control in this assay (IC_50_ = 240.00 ± 0.03 μg/ml). It was noted that 70% ethanolic extracts had higher inhibitory activity as compared to their corresponding water extracts. Our results demonstrated that the highest potential *α*-glucosidase inhibitory activity was shown by the ethanolic and water extracts of *A. nilotica* and *Z. spina-christi* with IC_50_ values ranging between 1.0–5.0 μg/ml, which were significantly higher than that of the positive control, Acarbose. In addition, the ethanolic extract of *M. annua, C. rotundus*, *B. papyrifera* and *G. alata* demonstrated strong enzymatic inhibition with IC_50_ values of 78.78 ± 3.19, 118.92 ± 1.01, 141.57 ± 1.54 and 191.66 ± 16.07 μg/ml, respectively. In case of water extract, *C. sinensis*, *C. proximus and G. alata*, had a slightly lower inhibitory activity compared to Acarbose with IC_50_ values of 260.0 ± 0.06, 280.0 ± 0.06 and 610.0 ± 0.02 μg/ml, respectively*.* Interestingly, *C. sinensis* and *C. proximus* were found to have their activity only in water extract, which may support  their traditional use as antidiabetic agents.

**Table 4:** *α*-glucosidase inhibitory activity of extracts

Scientific name	IC_50_ (μg/ml)^a^ value for *α*-glucosidase inhibition	
	70% Ethanol extract	Water extract
*A. precatorius*	–	–
*A. nilotica*	3.75 ± 0.62	1.33 ± 0.57
*B. linariifolia*	–	–
*B. papyrifera*	141.57 ± 1.54	–
*C. ipecacuanha*	–	–
*C. colocynthis*	–	–
*C. sinensis*	–	265.66 ± 5.13
*C. proximus*	–	282.37 ± 7.26
*C. rotundus.*	118.92 ± 1.01	–
*G. alata*	191.66 ± 16.07	623.99 ± 22.54
*M. annua*	78.78 ± 3.19	–
*P. glaucum*	–	–
*R. graveolens*	162.09 ± 52.08	–
*S. argel*	–	–
*T. bakis*	–	–
*T. foenum-graceum*	–	–
*V. madagascariensis*	–	–
*Z. spina-christi*	3.35 ± 0.61	5.49 ± 0.50
Acarbose^b^	240.00 ± 0.03	

^a^ Each value represents the mean ± SD (n = 3)

^b^Acarbose was used as a positive control

### Lipase inhibitory activity

In the current study, pancreatic lipase inhibitory activity was evaluated for all extracts, using Cetilistat as a positive control, with the IC_50_ value of 4.66 ± 0.05 μg/ml (Table 5). While the majority of the extracts demonstrated some extent of inhibitory activity against lipase enzyme, some of the plant extracts were particularly active. The ethanolic extracts of *A. nilotica*, *Z. spina-christi, S. argel*, *M. annua* and *B. papyrifera* exhibited a stronger inhibitory activity against pancreatic lipase enzyme compared to Cetilistat, with IC_50_ values of 1.65 ± 0.02, 1.86 ± 0.07, 2.12 ± 0.08, 4.22 ± 0.08 and 4.33 ± 0.07 μg/ml, respectively. Moreover, the water extracts of *A. nilotica*, *Z. spina-christi* and *C. sinensis* had a strong lipase inhibitory activity with IC_50_ values of 0.31 ± 0.01, 0.72 ± 0.01 and 4.51 ± 0.08 μg/ml, respectively.

**Table 5:** Pancreatic lipase inhibitory activity of extracts

Scientific name	IC_50_ (μg/ml)^a^ value for pancereatic lipase inhibition	
	70% Ethanol extract	Water extract
*A. precatorius*	17.55 ± 1.36	27.46 ± 1.58
*A. nilotica*	1.65 ± 0.02	0.31 ± 0.01
*B. linariifolia*	16.78 ± 0.31	18.12 ± 0.27
*B. papyrifera*	4.33 ± 0.07	46.19 ± 1.21
*C. ipecacuanha*	8.23 ± 0.12	22.1 ± 0.77
*C. colocynthis*	63.56 ± 1.6	20.66 ± 1.04
*C. sinensis*	29.82 ± 0.22	4.51 ± 0.08
*C. proximus*	16.69 ± 0.52	18.43 ± 0.28
*C. rotundus.*	8.16 ± 0.17	5.38 ± 0.42
*G. alata*	19.99 ± 0.77	23.76 ± 0.26
*M. annua*	4.22 ± 0.08	9.28 ± 1.81^*^
*P. glaucum*	119.91 ± 2.78	19.58 ± 0.17
*R. graveolens*	9.37 ± 0.42	48.03 ± 1.38
*S. argel*	2.12 ± 0.08	33.96 ± 0.29
*T. bakis*	17.75 ± 0.27	60.38 ± 0.88
*T. foenum-graceum*	244.15 ± 1.80	51.86 ± 2.67
*V. madagascariensis*	35.10 ± 5.56	98.66 ± 3.60
*Z. spina-christi*	1.86 ± 0.07	0.72 ± 0.01
Cetilistat^b^	4.66 ± 0.05	

^a^Each value represents the mean ± SD (*n* = 3)

^b^Cetilistat was used as a positive control of lipase inhibitory activity

### Cell genotoxicity/cytotoxicity activity

**Fig. 1:**
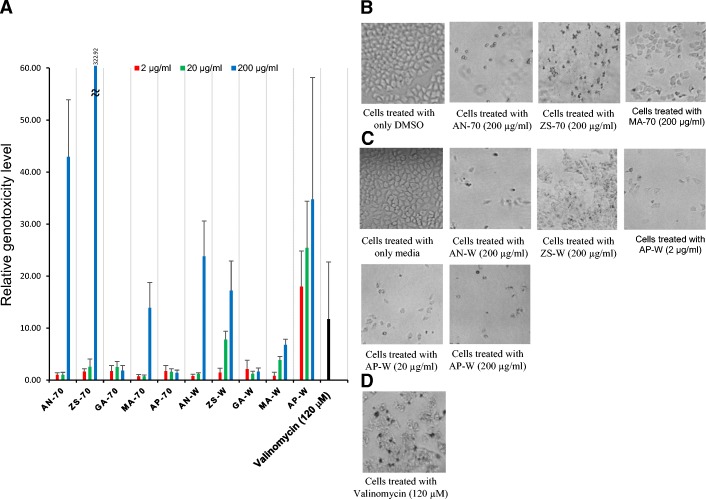
Imaging of genotoxicity of Sudanese medicinal plants in HeLa cells using the HCS DNA Damage Kit, (**a**) Chart showing the relative genotoxicity level of the 70% ethanol and water extracts of five Sudanese medicinal plants (AN: *Acacia nilotica*, ZS: *Ziziphus spina-christi*, GA: *Geigeria alata.* MA: *Martynia annua* AP: *Abrus precatorius*; For each extract, 70 represents 70% EtOH extract and W represents water extract), (**b**) Cells treated with 70% ethanolic extracts; (**c**) Cells treated with water extracts; (**d**) Positive control experiment, HeLa cells were treated with 120 μM valinomycin for 24 h at 37 °C/5% CO_2_. Imaging and analysis were performed using a BZ-X700. At 120 μM valinomycin, cells showed genotoxic effects as demonstrated by the positive pH2AX. The level of genotoxicity in the control experiment was set as 1.0. The fold change in signal between treated samples and control was > 3-fold for a parameter indicating genotoxicity

Based on the above results of the bioactivities screening; the 70% ethanol and water extracts of *A. nilotica*, *Z. spina-christi*, *M. annua, A. precatorius*, and *G. alata* were investigated for potential genotoxicity and cytotoxicity on HeLa cell line using HCS DNA Damage assay at various concentrations (2 μg/ml, 20 μg/ml, and 200 μg/ml) (Fig. 1a). As shown in Fig. 1b, the 70% ethanol extracts of *A. nilotica, Z. spina-christi*, and *M. annua* showed genotoxicity at concentrations > 20 μg/ml. In addition, a significant level of genotoxicity was observed at concentrations > 20 μg/ml for the water extracts of *Z. spina-christi* and 2 μg/ml for the water extracts of *A. precatorius*. Whereas, water extract of *A. nilotica* showed genotoxicity at concentrations > 20 μg/ml (Fig. 1c).

**Fig. 2:**
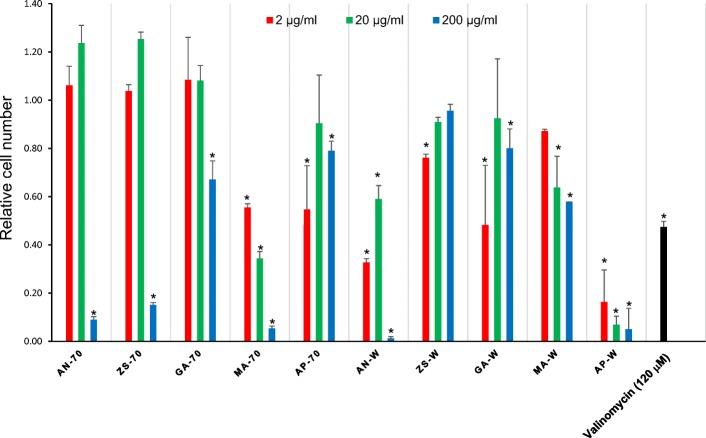
Imaging of cytotoxicity of Sudanese medicinal plants in HeLa cells using the HCS DNA Damage Kit. AN: *Acacia nilotica*, ZS: *Ziziphus spina-christi*, GA: *Geigeria alata.* MA: *Martynia annua* AP: *Abrus precatorius*; For each extract, 70 represents 70% EtOH extract and W represents water extract. As a control experiment, HeLa cells were treated with 120 μM valinomycin for 24 h at 37 °C/5% CO_2_. Imaging and analysis were performed using a BZ-X700. At 120 μM valinomycin, cells showed cytotoxic effects as demonstrated by the positive Image-iT® DEAD Green™ viability stain fluorescence

On the other hand, results showed a significant level of cytotoxicity in terms of depressing the HeLa cell viability of the 70% ethanol extracts of *A. nilotica* (200 μg/ml), *Z. spina-christi* (200 μg/ml), *G. alata* (200 μg/ml), *M. annua* (2, 20, 200 μg/ml), and *A. precatorius* (200 μg/ml). Similarly, the water extracts of *A. nilotica* (2, 20, 200 μg/ml), *G. alata* (200 μg/ml), *M. annua* (20, 200 μg/ml) and *A. precatorius* (2, 20, 200 μg/ml) exhibited a significant level of cytotoxicity as well (Fig. 2).

## Discussion

The increasing reliance on traditional herbal medicines as an approach to therapy has been observed worldwide. Herbal remedies are prepared traditionally as macerates, potions or decoctions using water as a solvent and given either as a mixture or as a single medicinal plant [10]. However, the use of these herbal remedies and medicinal plants without scientific validation of their claimed activity can be harmful and sometimes fatal. Accordingly, we designed this study with the aim of discovering potential and safe antidiabetic agents from plant origin and to validate the traditional uses of these plants as antidiabetic medicines.

In the current study, different parts of eighteen Sudanese medicinal plants were selected according to their traditional uses (Table 1), many of which are commonly used by Sudanese people to treat different ailments, including diabetes mellitus. Two solvents were used for extracting plant samples, 70% ethanol and water, which plays an important role in isolating different phytochemical from the plant and consequently different activities will be obtained [25, 30]. The use of solvents of different polarities has a direct association with the percentage yield of the extract. It was noted that the yield percentage of water extracted samples was relatively higher than that of 70% ethanol, which may be attributed to the higher solubility of carbohydrates and proteins in water than in ethanol [31]. Thereafter, the obtained extracts were screened for their potential antioxidant activity as well as, *α*-glucosidase inhibitory and pancreatic lipase inhibitory activities.

Oxidative stress occurs when production of oxidants or reactive oxygen species (ROS) exceeds local antioxidant capacity and it is thought to be associated with the pathogenesis of many diseases including diabetes mellitus [32]. Antioxidants are molecules that decelerate or quench free radical reactions and hence, delay or prevent cellular damage [33]. Medicinal plants are rich sources of secondary metabolites that act as natural antioxidant such as phenolic compounds (cinnamic acids, benzoic acids, flavonoids, proanthocyanidins, stilbenes, coumarins, lignans, and lignins), ascorbic acid and carotenoids [34, 35]. According to our results, *A. nilotica* showed the most potent antioxidant activity among other tested plants. There are several phytochemical studies on the pods of *A. nilotica,* revealed the presence of various polyphenolic compounds, such as isoquercetin, rutin, leucocyanidin, different galloylated derivatives of catechin, which possess free radical scavenging properties [36, 37]. Furthermore, the ethanolic extracts of *Z. spina-christi* and *A. precatorius* had relatively similar activity as the positive control, which is consistent with previous phytochemical studies that reported the presence of polyphenols, flavonoids, and tannins in both species and can be responsible for their potent antioxidant activities as well [38, 39]. Similarly, the ethanolic extracts of *M. annua* and *G. alata*, had moderate free radical scavenging activities. Kenwat et al. (2014) investigated the phytoconstituents of fruit oil of *M. annua* and reported the presence of flavonoids, tannins and phenolic compounds that may be attributed to the plant antioxidant activity [40]. To the best of our knowledge, this is the first screening study on *M. annua* species from Sudan [41]. A recent study by Zheleva-Dimitrova et al. (2016) investigated the phenolic acids contents of *G. alata* roots and leaves and identified chlorogenic acids that revealed antioxidant potentials [42]. Notably, the extracts obtained using 70% ethanol, illustrated stronger radical scavenging capacity than water extracts, which may be due to the ability of the organic/aqueous solvent to extract more phenolic compounds (a higher number of hydroxyl groups) that possess antioxidant activity, than extraction with water alone. Do et al. (2014) has reported a similar effect of extraction solvents on the radical scavenging activity, with stronger antioxidant activity in ethanolic/aqueous extracts compared to water extracts [43].

Oral antidiabetic (hypoglycemic) drugs such as Acarbose act by competitive and reversible inhibition of intestinal *α*-glucosidases, which slows down carbohydrate digestion and thus leads to a reduction of postprandial blood glucose levels [44]. As mentioned above, the strongest *α*-glucosidase inhibitory activity was shown by *A. nilotica* extracts*.* On the basis of the previous reports, it is possible that the presence of polyphenols, flavonoids, and tannins are responsible for the observed antidiabetic activity. Our results are also consistent with the in vivo study on the therapeutic effect of *A. nilotica* pod on diabetic rats, Enayat et al. (2012) has reported a significant reduction in blood glucose levels in the *A. nilotica* treated group in comparison to diabetic control rats [45], which support the *α*-glucosidase inhibitory activity of *A. nilotica* as a mechanism of lowering blood glucose levels. In addition, the strong *α-*glucosidase inhibitory activity of the ethanolic extract of *M. annua,* and *G. alata* suggest a correlation with their antioxidant activities due to the presence of bioactive compounds. For the best of our knowledge, it is the first report of the *α-*glucosidase inhibitory activity of the fruit of *M. annua.*

Inhibitors of pancreatic lipase such as Cetilistat, act by diminishing the absorption of dietary fats. Many reported secondary metabolites in plants, have already shown profound inhibition of pancreatic lipase enzyme such as phenolics, glycosides, saponins, and terpenes [46]. Our results showed that the majority of the tested extracts demonstrated some extent of inhibitory activity against lipase enzyme, particularly the extracts of *A. nilotica* and *Z. spina-christi.* This strongly suggests that these plants can be a source of potential leads for pancreatic lipase inhibition in order to treat and/or prevent obesity and other lifestyle-related diseases.

Prominently, during the evaluation of the bioactivities of the crude extracts, it is crucially important to consider the cytotoxicity testing of the bioactive extracts to exclude the toxic compounds that can impediment their therapeutic indications [47]. In the current study, 10 bioactive extracts from 5 plants species namely; *A. nilotica, Z. spina-christi, M. annua, A. precatorius,* and *G. alata* were selected for further investigations of their genotoxicity and cytotoxicity on HeLa cell line. Regarding the genotoxicity effect in comparison with the antioxidant activity, the IC_50_ values of *A. nilotica* and *G. alata* were non-genotoxic for both extracts along with the ethanolic extracts of *A. precatorius* and *Z. spina-christi*. Contrarily, the ethanolic extracts of *M. annua* and the water extracts of *A. precatorius* and *Z. spina-christi* were considered as genotoxic at their IC_50_ concentrations. Furthermore, *A. nilotica* and *Z. spina-christi* extracts were considered to be non-cytotoxic at their IC_50_ concentrations, in addition to the ethanolic extract of *A. precatorius* and the water extract of *G. alata.* In contrast, the ethanolic extracts *G. alata* and *M. annua,* along with the water extract of *A. precatorius* were found to be cytotoxic for HeLa cells at their activity concentrations.

On the other hand, in comparison with the IC_50_ values of the *α*-glucosidase inhibitory and lipase inhibitory activities of the extracts, it was found that both extracts of *A. nilotica* along with the ethanolic extract of *Z. spina-christi* were found to be non-genotoxic and non-cytotoxic at their IC_50_ values, which support the use of these bioactive crude extracts as safe anti-diabetic agents. Consequently, these findings recommend the use of these bioactive crude extracts as antioxidant, anti-diabetic, and anti-obesity agents under consideration to their toxicity levels. In addition, it necessitates further investigations for the bioactive extracts to identify the responsible compounds for the observed antioxidant, *α*­glucosidase, and lipase inhibitory activities.

## Conclusion

In the present study, eighteen of the Sudanese medicinal plants were screened for their potential antioxidant activity, *α*-glucosidase inhibitory and porcine pancreatic lipase inhibitory activities. Based upon the results, it could be concluded that *Acacia nilotica*, *Ziziphus spina-christi, Geigeria alata, Martynia annua, Abrus precatorius, Cordia sinensis* and *Boswellia papyrifera* exhibited an appreciable range of activity on the tested bioactivities, which highlights and supports their potential use as natural antidiabetic agents with strong antioxidant and anti-hyperlipidemic effects. Moreover, the genotoxicity and cytotoxicity assay findings suggest that although some of the tested bioactive extracts can be cytotoxic and/or genotoxic, however, their use within the safe concentration limits or as pure bioactive compounds can be of great benefit therapeutically. Our results will be a guide for the selection of plant species for further investigation in the discovery of new bioactive compounds from natural origin. Further supportive studies in chemical isolation and animal model experiments can be performed to confirm the in vitro antioxidant and enzymatic inhibition activities of the extracts.

## Abbreviations

4-MUFO: 4-methylumbelliferyl oleate
DMEM: Dulbecco’s Modified Eagle’s medium
DMSO: Dimethylsulfoxide
DPPH: 1,1-diphenyl-2-picrylhydrazyl
FCS: Fetal calf serum, BSA: Bovine serum albumin
MES: 2-morpholinoethanesulfonic acid, mono hydrate
PNGP: *P*-nitrophenyl glucopyranoside
PPL: Porcine pancreatic lipase
ROS: Reactive oxygen species
